# Therapeutic efficacy of CXCR3 blockade in an experimental model of severe sepsis

**DOI:** 10.1186/cc11642

**Published:** 2012-09-19

**Authors:** Daniela S Herzig, Yin Guo, Geping Fang, Tracy E Toliver-Kinsky, Edward R Sherwood

**Affiliations:** 1 Department of Anesthesiology, The University of Texas Medical Branch and Shriners Hospital for Children, 301 University Blvd, Galveston, TX, USA 77555; 2 Department of Microbiology and Immunology, The University of Texas Medical Branch 301 University Blvd, Galveston, TX, USA 77555; 3 Department of Biochemistry and Molecular Biology, The University of Texas Medical Branch 301 University Blvd,Galveston, TX, USA; 4 Department of Anesthesiology, Vanderbilt University Medical Center, 1211 Medical Center Drive, Nashville, TN, USA

## Abstract

**Introduction:**

In our previous studies we demonstrated that CXC chemokine receptor 3 (CXCR3) participates in the regulation of lymphocyte trafficking during cecal ligation and puncture (CLP)-induced sepsis. In this study, we evaluated the effects of treatment with anti-CXCR3 immunoglobulin (IgG) and antibiotics on outcome during septic shock caused by CLP.

**Methods:**

C57BL/6J mice were treated with neutralizing IgG against CXCR3 plus Primaxin either 24 hours prior to, 2 hours after or 6 hours after CLP. Control mice received nonspecific IgG plus Primaxin in the same regimen. Survival, core body temperature, bacterial clearance and systemic cytokine production were evaluated.

**Results:**

Our results show that treatment with anti-CXCR3 IgG plus Primaxin significantly improved survival when administered 24 hours prior to CLP (50% vs. 10%), 2 hours after CLP (55% vs. 10%) or 6 hours after CLP (55% vs. 25%) compared with mice receiving nonspecific IgG plus Primaxin. Treatment with anti-CXCR3 plus Primaxin 24 hours prior to CLP attenuated hypothermia and IL-6 and macrophage inflammatory protein 2 (MIP-2) production but did not alter bacterial clearance. Treatment with anti-CXCR3 IgG and Primaxin 2 hours after CLP did not improve bacterial clearance and systemic cytokine production compared with mice treated with IgG and Primaxin, whereas 6 hours after CLP the bacterial clearance and IL-6 and MIP-2 concentrations, both in plasma and peritoneal lavage fluid, were significantly improved in mice receiving anti-CXCR3 IgG and Primaxin compared with mice that only received nonspecific IgG and Primaxin.

**Conclusion:**

The results from this study indicate that neutralization of CXCR3 prior to, 2 hours after or 6 hours after the initiation of CLP-induced septic shock improves survival and attenuates CLP-induced inflammation and physiologic dysfunction.

## Introduction

CXC chemokine receptor 3 (CXCR3) is a G-protein coupled chemokine receptor that is activated by the CXC chemokine ligands CXCL9 (monokine induced by IFNγ), CXCL10 (interferon-inducible protein 10) and CXCL11 (interferon-inducible T-cell alpha chemoattractant) [1,2]. The CXCR3 ligands are produced by several cell types, primarily in response to type I interferons (IFNα/β) and IFNγ [1,3,4]. CXCR3 is an important regulator of natural killer (NK) lymphocyte, NK T lymphocyte and T-helper type 1 (Th1) lymphocyte trafficking in response to viral infection, allotransplantation, cancer and autoimmune diseases [4-11]. The CXCR3 ligands act redundantly or additively to regulate lymphocyte trafficking, depending on the disease process and tissue under study [1]. More recently, evidence has emerged that supports a role for CXCR3 activation in the pathogenesis of sepsis. High levels of CXCL10 have been observed in the plasma of septic patients, and plasma CXCL10 concentrations have been shown to parallel the severity of sepsis in humans [12,13]. Punyadeera and colleagues showed that increasing plasma CXCL10 concentrations were predictive of progression from sepsis to septic shock in critically ill patients [13]. In other clinical studies, plasma CXCL10 concentrations have been shown predictive of neonatal sepsis and systemic infection in infants with high sensitivity and specificity [14,15].

Our recent studies show that CXCR3 is an important regulator of NK cell trafficking during severe sepsis caused by cecal ligation and puncture (CLP) [16,17]. High concentrations of CXCL9 and CXCL10 were measured in peritoneal lavage fluid and plasma in the first 8 hours after CLP, and a gradient was noted such that CXCL9 and CXCL10 concentrations were higher in peritoneal lavage fluid than in plasma. In parallel, large numbers of CXCR3^+ ^NK cells were found to leave the spleen and blood prior to appearing in the peritoneal cavity, a phenomenon that was ablated in CXCR3-deficient mice and in mice treated with anti-CXCR3 IgG [16]. Peak NK cell recruitment was noted to occur between 8 and 16 hours after CLP. CXCR3 was also expressed by large numbers (>90%) of NK T cells and a subset of T lymphocytes, but those cell populations did not exhibit trafficking to the site of infection during the initial 8 to 16 hours after CLP. Compared with wildtype control mice, survival is improved in septic CXCR3-deficient mice and is associated with decreased systemic cytokine production and attenuated development of hypothermia [16]. The numbers of bacteria in peritoneal lavage fluid, blood and the lung were not generally different in CXCR3-deficent mice compared with controls. The improved outcomes observed in mice with CXCR3 deficiency therefore appear to be caused by attenuation of systemic inflammation and organ dysfunction.

Our previous studies examined outcome, systemic inflammation and bacterial clearance in CXCR3 knockout mice and in mice treated with anti-CXCR3 immunoglobulin prior to the initiation of sepsis. In the present study, the effect of CXCR3 blockade, when administered after the initiation of sepsis, was investigated. The goal of the study was to determine whether administration of anti-CXCR3 IgG after the initiation of sepsis will attenuate the pathobiology of sepsis and improve survival compared with mice treated with nonspecific IgG.

## Materials and methods

### Mice

C57BL/6J mice were purchased from the Jackson Laboratory (Bar Harbor, ME, USA). Mice were utilized in studies at 10 to 12 weeks of age. All studies were approved by the Institutional Animal Care and Use Committee at the University of Texas Medical Branch and complied with the National Institutes of Health Guide for the Care and Use of Experimental Animals.

### Cecal ligation and puncture

CLP was performed as described previously [16]. Briefly, mice were anesthetized with 2 to 3% isoflurane in oxygen. After shaving and aseptic preparation of the surgical site, a 1 to 2 cm midline incision was made through the abdominal wall; the cecum was identified and ligated with a 3-0 silk tie 1 cm from the tip. A double puncture of the cecal wall was performed with a 20-gauge needle. The incision was closed with Autoclips (Becton Dickinson, Sparks, MD, USA). Mice received intraperitoneal injection with 1 ml lactated Ringer's solution containing Primaxin (imipenem, a thienamycin antibiotic and cilistatin, an inhibitor of the renal dipeptidase, dehydropeptidase, 25 mg/kg; Merck & Co, Whitehouse Station, NJ, USA) at the time of CLP, 2 hours after CLP or 6 hours after CLP. In post-treatment experiments, mice received intravenous injection (0.1 ml) with either anti-CXCR3 IgG (clone CXCR3-173, 100 μg; eBioscience, San Diego, CA, USA) or isotype-matched, nonspecific IgG (100 μg; eBioscience) 2 hours or 6 hours after CLP. In pretreatment experiments, mice were treated with anti-CXCR3 IgG or nonspecific IgG 24 hours prior to CLP. All mice received buprenorphene (2.5 μg subcutaneously) 30 minutes before surgery and every 8 to 12 hours thereafter for analgesia. In survival studies, mice were observed over a 2-week period. Rectal temperature, cytokine concentrations and bacterial counts were performed on samples collected 18 hours after CLP.

### ELISA analysis

Heparinized blood was obtained by carotid laceration and plasma was harvested from centrifuged blood (2,000×*g *for 15 minutes). Lavage of the peritoneal cavity was performed with 3 ml sterile PBS. The IL-6 and macrophage inflammatory protein 2 (MIP-2) concentrations in peritoneal lavage fluid and plasma were measured using an ELISA according to the manufacturer's protocol (eBioscience; and R&D Systems, Minneapolis, MN, USA). Cytokine concentrations were determined by measuring optical density at 450 nm using a microtiter plate reader (Dynatech Laboratories, Chantilly, VA, USA). Previous studies from our laboratory, and others, show that the magnitude of systemic IL-6 and MIP-2 production during CLP-induced sepsis parallels the severity of physiologic dysfunction and mortality [16,17].

### Measurement of temperature and bacterial counts

Body temperature was measured by insertion of a rectal temperature probe immediately after induction of anesthesia with 2 to 3% isoflurane in oxygen. Previous studies from our laboratory, and others, show that the degree of hypothermia during CLP-induced sepsis parallels the severity of physiologic dysfunction and mortality [16,17]. Bacterial counts were performed on blood, peritoneal lavage fluid and the lung. Samples of plasma and peritoneal lavage fluid were obtained as described above. Lung tissue was harvested by thoracotomy under aseptic conditions, weighed and homogenized in sterile PBS to achieve a final concentration of 11 mg tissue per milliliter of saline. Samples were serially diluted in sterile saline and cultured on tryptic soy agar pour plates. Plates were incubated (37°C) for 24 to 48 hours and colony counts were performed by direct visualization.

### Statistical analysis

All data were analyzed using GraphPad Prism software (GraphPad, San Diego, CA, USA). Data from multiple group experiments were analyzed using one-way analysis of variance followed by a *post hoc *Tukey's test to compare groups. Paired data were analyzed using a paired *t *test. For measurements of bacterial colony-forming, groups were compared using a nonparametric Kruskal-Wallis test followed by a *post hoc *Dunn's test. Survival data were analyzed using the log-rank test. *P *<0.05 was considered statistically significant for all experiments. All values are presented as the mean ± standard error of the mean, except for bacterial counts, for which median values are designated.

## Results

### Treatment with anti-CXCR3 after CLP improves survival and attenuates sepsis-induced hypothermia

**Figure 1:**
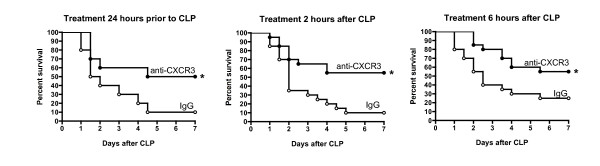
**Treatment with anti-CXCR IgG improves survival during cecal ligation and puncture-induced sepsis**. Mice were treated with anti-CXC chemokine receptor 3 (anti-CXCR3) IgG (100 μg) or nonspecific IgG (100 μg) 24 hours prior to (*n *= 10 mice per group), 2 hours after (*n *= 20 mice per group) or 6 hours after (*n *= 20 mice per group) cecal ligation and puncture (CLP). Mice were followed for survival over a 2-week period. *P <0.05 compared with nonspecific IgG.

Treatment with anti-CXCR3 IgG significantly improved survival when administered 24 hours prior to (50% vs. 10%), 2 hours after (55% vs. 10%) or 6 hours after (55% vs. 25%) CLP compared with mice treated with nonspecific IgG (Figure 1). All mice that survived beyond the 7-day time point were alive and did not show signs of morbidity 2 weeks after CLP.

**Figure 2:**
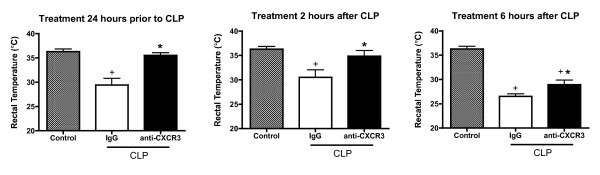
**Effect of anti-CXCR3 IgG treatment on core temperature**. Mice were treated with anti-CXC chemokine receptor 3 (anti-CXCR3) IgG (100 μg) or nonspecific IgG (100 μg) 24 hours prior to (*n *= 10 mice per group), 2 hours after (*n *= 10 mice per group) or 6 hours after (*n *= 13 mice per group) cecal ligation and puncture (CLP). Rectal temperature was measured 18 hours after CLP. Mice designated as control represent nonseptic mice. *P <0.05 compared with nonspecific IgG, ^+^*P *<0.05 compared with control.

The development of hypothermia is a predictor of sepsis-induced physiologic dysfunction in mice [16]. Rectal temperature was significantly lower in all mice that underwent the CLP procedure and were treated with nonspecific IgG compared with control nonseptic mice (Figure 2). Rectal temperature was significantly higher in mice treated with anti-CXCR3 IgG compared with mice treated with nonspecific IgG, regardless of whether treatment was initiated 24 hours before or 2 to 6 hours after CLP (Figure 2).

### Effect of anti-CXCR3 treatment on bacterial burden

**Figure 3:**
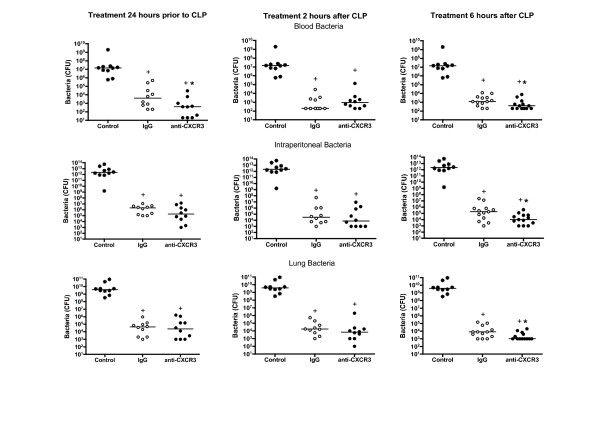
**Effect of anti-CXCR3 IgG treatment on bacterial burden**. Mice were treated with anti-CXC chemokine receptor 3 (anti-CXCR3) IgG (100 μg) or nonspecific IgG (100 μg) 24 hours prior to, 2 hours after or 6 hours after cecal ligation and puncture (CLP). Blood and peritoneal lavage fluid were collected 18 hours after CLP and bacterial colony-forming units (CFU) were measured. Mice designated as control represent septic mice that did not receive antibiotic treatment. *n *= 10 to 13 mice per group. *P <0.05 compared with nonspecific IgG, ^+^*P *<0.05 compared with control.

Mice were treated with Primaxin at the time of CLP or at the time of anti-CXCR3 administration in cases where a post-treatment protocol was used. Treatment of mice with Primaxin decreased bacterial counts in the blood, peritoneal cavity and lung by >99% compared with control septic mice that did not receive antibiotic treatment (Figure 3). Bacterial counts after treatment with anti-CXCR3 IgG and Primaxin were not significantly different from those in mice treated with nonspecific IgG and Primaxin, except in the 6-hour post-treatment group where a small but statistically significant decrease in bacterial counts was observed in the blood, peritoneal cavity and lung (Figure 3).

### Effect of anti-CXCR3 treatment on proinflammatory cytokine production

**Figure 4:**
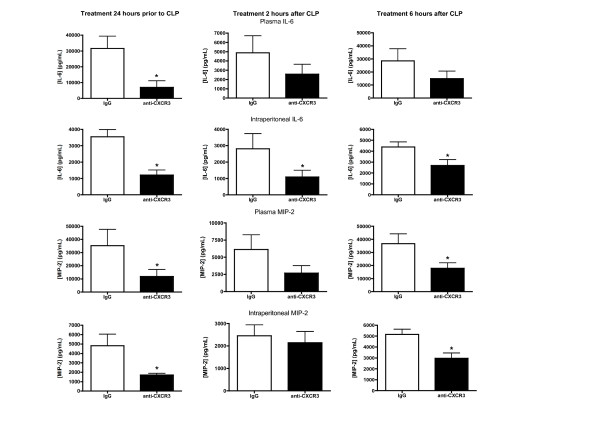
**Effect of anti-CXCR3 IgG treatment on cytokine production**. Mice were treated with anti-CXC chemokine receptor 3 (anti-CXCR3) IgG (100 µg) or nonspecific IgG (100 µg) 24 hours prior to (*n *= 10 mice per group), 2 hours after (*n *= 10 mice per group) or 6 hours after (*n *= 13 mice per group) cecal ligation and puncture (CLP). Plasma and peritoneal lavage fluid were harvested 18 hours after CLP for measurement of IL-6 and macrophage inflammatory protein 2 (MIP-2) concentrations by ELISA. *P <0.05 compared with nonspecific IgG.

Treatment of mice with anti-CXCR3 IgG 24 hours prior to CLP caused significant attenuation of IL-6 and MIP-2 concentrations in the blood and peritoneal lavage fluid compared with mice treated with nonspecific IgG (Figure 4). In the 2-hour and 6-hour post-treatment groups, IL-6 concentrations were significantly lower in the peritoneal lavage fluid from anti-CXCR3-treated mice than in mice treated with nonspecific IgG. A trend toward lower IL-6 concentrations was also observed in plasma from mice receiving treatment with anti-CXCR3 2 or 6 hours after CLP but the differences were not statistically significant. Concentrations of MIP-2 in the peritoneal lavage fluid and plasma were significantly lower in mice treated with anti-CXCR3 IgG 24 hours prior to CLP, but were not significantly different when comparing mice treated with anti-CXCR3 IgG 2 hours after CLP compared with those treated with nonspecific IgG (Figure 4). In the 6-hour post-treatment group, plasma and intraperitoneal IL-6 concentrations were significantly lower in mice treated with anti-CXCR3 IgG than in those treated with nonspecific IgG (Figure 4).

## Discussion

The present study shows that initiation of CXCR3 blockade, in combination with a broad-spectrum antibiotic, 2 hours or 6 hours after CLP provides a significant survival benefit compared with antibiotic treatment alone. Our previous studies examined outcome, systemic inflammation and bacterial clearance in CXCR3 knockout mice and in mice treated with anti-CXCR3 immunoglobulin prior to the initiation of sepsis. The findings of the present study extend our previous observations by showing the potential of CXCR3 blockade as a therapeutic approach to decrease the severity of sepsis during its acute phase. The survival benefit observed in mice treated with anti-CXCR3 IgG 2 hours or 6 hours after the initiation of severe sepsis was similar to that seen in mice treated with anti-CXCR3 24 hours prior to CLP and was associated with attenuation of sepsis-induced hypothermia and systemic cytokine production. Bacterial clearance was not markedly affected by CXCR3 blockade. Significant differences in bacterial colony-forming units were not observed at most time points evaluated in this study. However, a small, but statistically significant, difference in bacterial colony-forming units was observed in mice treated with anti-CXCR3 IgG 6 hours after CLP. Yet the difference in bacterial burden in the 6-hour post-treatment group was not associated with major differences in survival, core temperature or systemic cytokine production compared with mice treated with anti-CXCR3 IgG 24 hours before or 2 hours after CLP. The beneficial effects of CXCR3 blockade therefore do not appear to be dependent on augmentation of bacterial clearance mechanisms.

Few studies have examined the effects of CXCR3 blockade during sepsis. Most prior efforts aimed at targeting CXCR3 for therapeutic benefit have focused on chronic autoimmune diseases such as multiple sclerosis, inflammatory bowel disease and rheumatoid arthritis [5,18-20]. Other investigators have shown that CXCR3 blockade is beneficial in prolonging allograft survival in experimental models of organ transplantation [21]. Most early studies employed blocking antibodies against CXCR3 or its ligands as the primary strategy to antagonize CXCR3. More recent research has resulted in the development of nonpeptidergic, small molecular weight CXCR3 inhibitors [22-25]. The (aza)quinazolinone class of CXCR3 antagonists are the most promising, with AMG487 being the most extensively developed [22]. AMG487 has been shown to decrease T-cell migration and attenuate disease severity in experimental models of pulmonary fibrosis and collagen-induced arthritis [22]. Clinical trials showed that AMG487 was relatively well tolerated but did not have efficacy in patients with psoriasis. Studies are currently underway to modify the structure of AMG487 to decrease drug metabolism and minimize drug accumulation in humans. Whether nonpeptidergic CXCR3 inhibitors are efficacious in experimental models of sepsis remains to be determined. However, the present study shows that antibody-based CXCR3 blocking approaches are effective in the CLP model of septic shock and opens the possibility that other blocking strategies may be efficacious.

CXCR3 is expressed predominantly by NK lymphocytes, NK T lymphocytes and Th1 lymphocytes. Previous studies have shown that Th1 lymphocytes play an important role in the pathogenesis of chronic autoimmune diseases [26-28]. The effects of CXCR3 blockade in autoimmune diseases are therefore probably mediated through inhibition of Th1 lymphocyte trafficking to sites of chronic inflammation. However, our previous studies show that trafficking of CXCR3^+ ^T lymphocytes into the peritoneal cavity is minimal in the initial 16 hours after CLP and that CXCR3 deficiency or blockade does not alter T-cell trafficking into the primary site of infection during CLP-induced sepsis [16]. Likewise, we reported that the numbers of intraperitoneal CXCR3^+ ^NK T cells did not change during the course of CLP-induced septic shock. Yet large numbers of CXCR3^+ ^NK cells were found to migrate into the peritoneal cavity within 4 to 8 hours after CLP. The entry of CXCR3^+ ^NK cells into the peritoneal cavity paralleled a decline in splenic and blood NK cell numbers, and both alterations were ablated in CXCR3-deficient mice and in mice treated with anti-CXCR3 IgG. The trafficking of NK cells into the infected and inflamed peritoneal cavity coincided with elevations in CXCL9 and CXCL10 concentrations, with CXCR3 ligand concentrations being higher in the inflamed peritoneal cavity than in plasma. CXCR3-deficient mice and mice treated with anti-CXCR3 IgG showed improved survival, decreased systemic cytokine production and attenuated physiologic dysfunction compared with control mice. These observations imply that CXCR3-directed NK cell trafficking contributes to the pathogenesis of septic shock. At this point, however, that relationship is associative. Further work is needed to fully define how CXCR3 is activated and mediates inflammatory and physiological changes during severe sepsis.

NK cells and NK T cells are known to contribute to systemic inflammation and physiologic dysfunction during sepsis and polytrauma [17,29-31]. However, the mechanisms by which CXCR3 alters NK cell and NK T-cell function during sepsis and the systemic inflammatory response syndrome is not completely understood. NK cells do not uniformly express CXCR3. The proportion of CXCR3^+ ^NK cells varies between 20 and 60% in various tissues, with the peritoneal cavity, spleen, bone marrow, lung and liver representing the major NK cell-containing organs [32]. Murine CXCR3^+ ^NK cells have significant migratory potential and the capacity to secrete IFNγ and TNFα. In that regard, they are functionally similar to human CD56^bright ^NK cells. Like human CD56^dim ^NK cells, mouse CXCR3^- ^NK cells have low migratory potential, express high levels of CD11b, possess significant cytotoxic activity and secrete less IFNγ and TNFα compared with CXCR3^+ ^NK cells. Interestingly, our previous studies show that not all CXCR3^+ ^NK cells migrate to the site of infection after CLP [16]. A subset of CXCR3^+ ^NK cells remains in the spleen. In addition, large numbers of CXCR3^+ ^NK cells are present within the liver, lung and bone marrow and their numbers do not change significantly during CLP-induced sepsis. Our previous studies show that CXCR3 expression by hepatic NK cells decreases within 4 hours of CLP and continues to decline during the first 16 hours [16]. Likewise, the numbers of hepatic NK T cells, of which >90% are CXCR3^+^, do not change after CLP but CXCR3 expression by hepatic NK T cells declines after CLP in a pattern similar to that observed for liver NK cells [16]. The activation of NK cells and NK T cells is known to cause internalization and downregulation of CXCR3 [2]. Hepatic CXCR3^+ ^NK cells and NK T cells are therefore likely to become activated during CLP-induced sepsis but do not migrate out of the liver. The activation of NK T cells after CLP may facilitate intrahepatic trafficking since previous studies have shown that activation of CXCR3 functions to regulate the migration of NK T cells within the hepatic sinusoids during periods of bacterial infection [33]. However, whether hepatic NK cells and NK T cells contribute to systemic inflammation and physiologic dysfunction during septic shock and whether CXCR3 contributes to the activation of those cell populations remain to be determined.

## Conclusions

This study shows that inhibition of CXCR3 activation using blocking antibodies is efficacious in reducing the severity of CLP-induced sepsis. Treatment with anti-CXCR3 IgG was effective in prolonging survival when administered up to 6 hours after CLP. The beneficial effect is primarily associated with decreased systemic cytokine production and attenuated physiologic dysfunction and appears to be independent of bacterial clearance mechanisms. Further studies are needed to fully define the protective mechanisms of CXCR3 blockade during sepsis and to determine its value as an approach to limit injury and improve outcome during sepsis.

## Key message

• Treatment with anti-CXCR3 IgG up to 6 hours after CLP will decrease systemic cytokine production, attenuate physiologic dysfunction and improve survival in a clinically relevant experimental model of severe sepsis.

## Abbreviations

CLP: cecal ligation and puncture; CXCL: CXC chemokine ligand; CXCR3: CXC chemokine receptor 3; ELISA: enzyme-linked immunosorbent assay; IFN: interferon; IL: interleukin; MIP-2: macrophage inflammatory protein 2; NK: natural killer; PBS: phosphate-buffered saline; Th1: T helper type 1; TNF: tumor necrosis factor.

## Competing interests

The authors declare that they have no competing interests.

## Authors' contributions

DSH made major contributions to the design and interpretation of experiments and was actively involved in all experimental components of the study. YG was actively involved in all experiments performed including surgical procedures and cytokine measurements. GF was actively involved in performing microbiological analyses and surgical procedures, and was instrumental in interpreting findings and troubleshooting technical aspects of the study. TET-K was actively involved in the design and interpretation of experiments. ERS is the principal investigator and is responsible for all conceptual and technical aspects of this study. All authors read and approved the final manuscript.
